# Socio-demographic and clinical features of Irish iatrogenic hepatitis C patients: a cross-sectional survey

**DOI:** 10.1186/1471-2458-9-323

**Published:** 2009-09-07

**Authors:** Olivia McKenna, Caitriona Cunningham, Catherine Blake

**Affiliations:** 1 UCD School of Physiotherapy and Performance Science, University College Dublin, Belfield, Dublin 4, Ireland; 2 Department of Physiotherapy, Mid-Western Regional Hospitals, Dooradoyle, Limerick, Ireland

## Abstract

**Background:**

A discrete sub-group of iatrogenically-acquired hepatitis C virus (HCV)-infected individuals exists in the Irish population on whom limited current research data is available. The aim of this study was to establish a current profile of the socio-demographic and clinical characteristics of the Irish iatrogenic hepatitis C population and to determine factors predicting symptoms experienced.

**Methods:**

An anonymous, national, cross-sectional survey was conducted to explore this populations' self-reported health and social attributes. Data were collected on 290 respondents.

**Results:**

Mean time since infection was 26 years. Eighty-four percent (n = 237) of respondents were female (mean age = 55.9 ± 9.6 years). Hepatic and extra-hepatic symptoms were common (62% and 99% respectively). Fatigue and pain were frequent complaints while 89% reported diagnosed co-morbid disease. On logistic regression, female gender, age and co-morbid disease emerged as independent predictors of self-reported symptoms.

**Conclusion:**

This study describes the current status of the iatrogenically infected patient cohort in Ireland, adding to existing knowledge regarding the clinical course and consequences of HCV infection. Changing healthcare needs were shown by comparison with earlier surveys in this same population, in terms of disease progression, development of co-morbid disease and ageing.

## Background

Hepatitis C is a major public health problem. In 2000 the WHO calculated that there were more than 170 million chronic carriers worldwide (3% of world population) with 3-4 million persons infected each year [1]. The late effects of HCV infection include cirrhosis and hepatocellular carcinoma, with reports that 27% and 25% of HCV infected people progress to these respectively [2]. Hepatitis C is predominantly transmitted by means of exposure to infected blood products and most new cases are related to intravenous drug use (IVDU) [3].

Complete figures for the prevalence of HCV infection in Ireland are not available [4], but annual incidence is now being recorded with 1,572 new cases reported in 2007 [5]. In Ireland today new cases of hepatitis C may be the result of HCV transmission through any of the recognised infection routes (62% of those recorded in 2007 were related to IVDU [6] (63% of whom were male [5])). A discrete sub-group of people infected through transfusion with contaminated blood products however exists in the Irish HCV population. The National Hepatitis C Database recently reported that, during the period 1970-1994, over 1600 individuals have been infected with HCV through this mechanism, including people with haemophilia, kidney disease and recipients of infected anti-D serum and blood transfusions [7].

In Ireland, epidemiologic evaluation identified a single HCV-infected individual as the source of virally contaminated anti-D administered to thousands of rhesus negative women during 1977 and 1978 [8]. This discovery provoked a health care crisis for the blood transfusion services and led to the establishment of a national screening programme in February 1994. In 1997 the Tribunal of Inquiry into the Blood Transfusion Service Board (BTSB), found that the primary cause of contamination of anti-D with HCV was a breach of the BTSB's own standards for donor selection and procedures [9]. A "targeted lookback" and subsequently an "optional" screening programme was then introduced for transfusion recipients in 1995 which identified a transfusion component and coagulation factor transmitted hepatitis C infection [10]. The Irish iatrogenic HCV cohort thus represents a homogeneous group with a common infection source and ethnic origin. Female predominance and a mean age at infection of 43-46 years are additional unique attributes of this subsection of the Irish HCV population [8,11,12].

Hepatitis C incurs considerable social, physical and mental health needs in those living with the disease [13,14]. In addition to hepatic disease and its associated symptoms which are well described [15-17] there are important extra-hepatic manifestations [16] which cause significant morbidity and impairment. Indeed it is these extra-hepatic manifestations which dominate the symptomatology of the condition and impair quality of life [16]. Extra-hepatic manifestations of HCV may involve multiple body systems [18] however there is a preponderance of rheumatic (arthralgia, myalgia, parasthesia) and cutaneomucous (pruritis, sicca syndrome, Raynaud's phenomenon) symptoms [16]. Cacoub et al. [19] reported that the most frequent extra-hepatic manifestations of HCV were general symptoms (arthralgia, myalgia and parasthesia), however these were shown to be independently associated with other extra-hepatic manifestations such as vasculitis, purpura, psoriasis and abnormal creatinine levels. Age, female sex and extensive liver fibrosis are reported as the most frequent risk factors for the presence of clinical and biologic extra-hepatic manifestations [19]. Co-morbid disease (e.g., obesity, diabetes, depression) is emerging as an important and relevant factor in HCV progression, symptom experience and general well-being [20-22]. Painful medical co-morbidities (e.g., arthritis) and depressed mood requiring treatment have been associated with greater levels of disability [20,23,24].

Much research has been done internationally in the hepatitis C population predominantly investigating the effectiveness of different therapies [25-28], quality of life issues [29-31], clinical symptoms [21,32,33], and prognosis [8,34]. Given the availability of the unique homogeneous cohort of iatrogenically infected individuals, research in Ireland has focused mainly on the genetic and biomolecular aspect of the condition [35-37]. Some research has examined the clinical symptoms and psychological aspects of the disease [8,11,12,24,38-41]. Given that there is a long latent period between HCV exposure and the emergence of chronic liver disease (at least 2 decades [17]), the importance of longitudinal follow-up has been acknowledged. The National Hepatitis C Database was created to gather information on this group of iatrogenically infected patients on an ongoing basis and it issued a report in 2007 based on data collected in 2005 and 2006 from medical chart reviews [7].

The circumstances surrounding the infection of members of the Irish population with contaminated blood and blood products renders this group a unique cohort for study. This, combined with the limited current research in the Irish cohort in terms of health and social factors highlights a deficit in the literature. The current study did not seek to duplicate data in the National Hepatitis C Database but sought to bring a more personal perspective of transfusion-related hepatitis C through the use of self-report data.

### Aim

The primary aim of this study was to describe the self-reported health and social attributes of patients with past or current exposure to iatrogenically transmitted hepatitis C infection in the Irish population. The specific aims addressed were:

1. To describe the socio-demographic characteristics of the iatrogenic hepatitis C population in Ireland approximately 30 years after the first recorded contamination

2. To establish the prevalence of co-morbid conditions in the group

3. To explore self-reported clinical manifestations of the disease

4. To explore factors predicting the most prevalent symptoms in this cohort.

## Methods

### Design

An anonymous, national, cross-sectional survey of patients with iatrogenic hepatitis C infection was undertaken, using a self-completed postal questionnaire. Participants were recruited during the period from November 2005 to March 2006.

### Study Population

Adult individuals aged 18 years and older, who contracted hepatitis C through contaminated, state-provided, blood products, were eligible for inclusion. In the absence of a complete national database at the time of data collection, the membership lists of the four main patient advocacy groups in the Republic of Ireland were selected as the most complete sampling frame, representing people with declared iatrogenic HCV infection. A population of one thousand and forty five was identified. Patients thus could be classified into: (i) anti-D, (ii) blood transfusion, (iii) haemophilia and (iv) kidney disease exposure groups. Seventy-two percent were members of Positive Action, 24% from Transfusion Positive, 2% were members of the Irish Kidney Association and 2% were from the Irish Haemophilia Society.

### Questionnaire

A customised survey using closed and open-ended questions was prepared. The process for questionnaire development followed recommended guidelines for survey construction [42] and involved consultation with the patient advocacy groups and a panel of experts (health professionals and patients) thus ensuring that acceptability and content validity of the questionnaire were assured. Data were collected on socio-demographic variables (age, gender, marital status, family circumstances and employment status) in addition to clinical history and symptoms (co-morbidity, date of infection, date of diagnosis, current antiviral therapy, symptoms (including site of pain if relevant), and respective healthcare management). Socio-demographic and employment-related questions were modelled on the labour force and national household surveys [43,44]. Clinical and symptom-related data were derived from self-report on customised questions based on clinical knowledge of hepatitis C and previous published research [15,19,45,46]. The burden of co-morbidity was measured using both a simple count of co-morbidity and the Charlson Co-morbidity Index (CCI) [47]. The CCI is a weighted index that takes into account the number and seriousness of co-morbid disease, with a higher number indicating more serious burden.

### Procedure

Ethical approval was granted by the local Regional Hospital Ethics Committee. Confidentiality issues precluded access by the research team to patients and their health records therefore the assistance of the four relevant patient advocacy groups was sought. Questionnaires were forwarded to the subjects by the advocacy groups accompanied by an information leaflet, a cover letter and a stamped addressed envelope. Reminder letters were forwarded to all recipients 3 weeks after the initial posting. Consent to participate was implied by means of reply and the respondent remained anonymous at all times. Returned data were transcribed on to a computerised data collection sheet which was stored securely. Colour coding of questionnaires allowed anonymous responses to be distinguished by patient group.

### Analysis

Data were analysed using SPSS (Statistical Package for the Social Sciences) version 12.0.1. Quantitative data were summarised using descriptive statistics. Relationships between variables were explored using Chi Square (χ^2^) and Fishers tests for categorical data. Independent t tests and one way ANOVA were used for ratio and interval data. Categorical and continuous data were re-categorised and recoded as appropriate. The International Labour Office (ILO) criteria [44] were applied to determine the number inactive in the labour force, the number of active labour force participants and employment rates. The ILO calculates employment rates by expressing the number employed as a percentage of those active (employed and unemployed) in the labour force. Use of this method facilitated direct comparison with national statistics, thus permitting the effects of HCV to be interpreted with reference to general Irish population norms. All symptoms reported by this population were perceived as being associated with hepatitis C in the absence of another definitive cause. Symptoms were categorised according to body structure involvement. Those relating to hepato-gastro-intestinal origins were termed hepatic and those relating to other structures were classified as extra-hepatic. Calculations for measures of disease duration and time elapsed since diagnosis, were made based on subtraction of the stated year from January 1^st ^2006 (mid-point of data collection period). Multivariate binary logistic regression analysis was then conducted on these categorical variables using a standard enter method to determine independent predictors of the most commonly identified symptom clusters which emerged. Findings of univariate analysis were used to build the regression models, commonly associated factors with these symptoms as reported in the literature were also considered to inform the content of the regression model. Haemophilia and mood disorders, relevant medical conditions to the study cohort were included as separate entities in the regression models, given that they were not accounted for in the calculation of the CCI [47] score. For significance level testing the p value was set at p < 0.05. Ninety-five percent confidence intervals for proportions and means were calculated using methods outlined by Altman et al. [48] and Confidence Interval Analysis Software version 2.1.2. [49].

## Results

### Sample Characteristics

**Figure 1:**
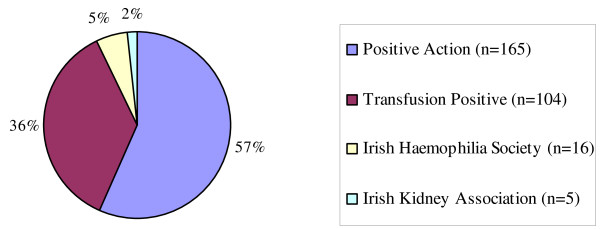
**Patient Group Representation**.

In total, 290 (28%) questionnaires were returned. Responses were received from members of all the patient advocacy groups (Figure 1).

### Socio-demographic Features

The main socio-demographic attributes for the total group and each of the sub-groups are listed in table 1. Of the total respondents 44 (16%) were male and 237 (84%) were female with a mean age of 55.9 ± 9.6 years, ranging from 20-80 years (median = 56 years). Twenty four out of the 32 counties in the island of Ireland were represented in the responses, with the highest response rate coming from Dublin with 35% (n = 83) of replies. Approximately 99% of the sample population were of Irish nationality.

**Table 1:** Iatrogenic Hepatitis C - Socio-demographic Features.

***Variable***	**Total** **(n = 290)**	**Positive Action** **(n = 165)**	**Transfusion Positive** **(n = 104)**	**Irish Haemophilia Society** **(n = 16)**	**Irish Kidney Association** **(n = 5)**
	**Mean (Sd)**	**Mean (Sd)**	**Mean (Sd)**	**Mean (Sd)**	**Mean (Sd)**
**Age **- (years)	55.9 (9.6)	55.8 (6.8)	57.9 (11.8)	46.2 (12.8)	52.2 (8.0)
					
**Gender **(n = 281)	**n(%)^a^**	**n(%)^a^**	**n(%)^a^**	**n(%)^a^**	**n(%)^a^**
Male	44 (16)	0 ( 0)	27 (27)	15 (94)	2 ( 40)
Female	237 (84)	161 (100)	72 (73)	1 ( 6)	3 ( 60)
**Education**(n = 271)					
Primary	59 (22)	32 ( 21)	25 (26)	2 (13)	0 ( 0)
Secondary	143 (53)	86 ( 55)	47 (49)	6 (40)	4 ( 80)
Tertiary	69 (25)	37 ( 24)	24 (25)	7 (47)	1 ( 20)
**Marital Status (**n = 279)					
Married	215 (77)	132 ( 83)	72 (74)	9 (56)	2 ( 40)
Not Married	64 (23)	28 ( 17)	26 (26)	7 (44)	3 ( 60)
**Dependents **(n = 263)					
Yes	103 (39)	65 ( 43)	32 (35)	5 (33)	1 ( 20)
No	160 (61)	86 ( 57)	60 (65)	10 (67)	4 ( 80)
**Employment Status**(n = 95)					
Employed	38 (40)	17 ( 35)	13 (38)	8 (80)	0 ( 0)
Unemployed	57 (60)	31 ( 65)	21 (62)	2 (20)	3 (100)

^a ^Where missing responses, percentages were calculated out of available responses

Further analysis of these findings found that females were older, with a mean age of 56.9 ± 8.3 years by comparison with 50.7 ± 13.5 years in males (t = 3.0, df = 49.3, 95% CI = 2.0-10.5, p = 0.004). Only 42% of the cohort was eligible to be active participants in the labour force as defined by the International Labour Office (ILO) Classification, the other 58% being retired, working in the home or students. Of the ninety-five eligible to work only 38 (40%) were in employment, of whom 59% (n = 22) were female. Permanent sickness or disability was cited by 52 individuals (91%) of the unemployed cohort as a reason for unemployment.

### Clinical Features

The mean time since diagnosis was 11.4 ± 3.6 years (median 12, range 3-29 years) with a mean time since infection of 26.3 ± 5.4 years (median 29, range 10-44 years) for the total group. Details of virus status and duration of infection are summarised in table 2. Over 43% (n = 125) of the total sample (n = 290) were virus (PCR) positive (i.e., had evidence of current infection). The figures quoted in table 2 excluded missing data. Of those who were virus (PCR) negative (evidence of resolved infection) (n = 61), 55.7% (n = 34) were virus negative following antiviral treatment. One percent (n = 3) of respondents reported undergoing antiviral treatment for hepatitis C at the time of the survey. Interestingly the majority of the respondents were unsure of their genotype. Over 62% (n = 180) either left the question unanswered or stated that they did not know what their genotype was. Of those respondents who were aware of their genotype (n = 110), genotype 1, 1a and 1b accounted for 71% (n = 78), 15% (n = 17) had genotype 2, while 12% (n = 13) had genotype 3. The remaining 2% (n = 2) of respondents reported types 4 and 5.

**Table 2:** Iatrogenic Hepatitis C - Clinical Features, Co-morbid Disease and Symptoms

***Variable***	**Total** **(n = 290)**	**Positive Action** **(n = 165)**	**Transfusion Positive** **(n = 104)**	**Irish Haemophilia Society** **(n = 16)**	**Irish Kidney Association** **(n = 5)**
	**Mean (Sd)**	**Mean (Sd)**	**Mean (Sd)**	**Mean (Sd)**	**Mean (Sd)**
Duration of Infection	26.3 (5.4)	27.9 (3.9)	23.5 (6.5)	26.1 (5.4)	29.5 (3.5)
Time Since Diagnosis	11.4 (3.6)	11.9 (3.7)	10.5 (3.3)	13.6 (2.4)	9.3 (1.3)
	**n (%)^a^**	**n (%)^a^**	**n (%)^a^**	**n (%)^a^**	**n (%)^a^**
**Virus Status**(n = 186)					
PCR Positive	125 (67)	74 (76)	42 (55)	6 ( 60)	3 (100)
PCR Negative	61 (33)	23 (24)	34 (45)	4 ( 40)	0 ( 0)
**Symptoms**					
Hepatic (n = 275)	169 (62)	95 ( 61)	63 (64)	6 ( 38)	5 (100)
Extra-hepatic (n = 278)	275 (99)	159 (100)	95 (97)	16 (100)	5 (100)
Fatigue (n = 274)	241 (88)	139 ( 90)	86 (88)	12 ( 75)	4 ( 80)
Pain (n = 252)	243 (96)	147 ( 97)	80 (95)	12 (100)	4 ( 80)
Sleep Disturbance (n = 274)	197 (72)	111 ( 72)	75 (77)	7 ( 44)	4 ( 80)
Itching (n = 274)	132 (48)	83 ( 54)	44 (45)	4 ( 25)	1 ( 20)
Abdominal Swelling (n = 273)	113 (41)	71 ( 46)	35 (36)	4 ( 25)	3 ( 60)
**Co-morbid Disease**(n = 286)					
Cardiovascular Disorder	144 (50)	88 ( 54)	48 (47)	3 ( 19)	5 (100)
Musculoskeletal Disorder	89 (31)	54 ( 33)	33 (32)	2 ( 13)	0 ( 0)
Mood Disorder	136 (48)	81 ( 50)	47 (46)	4 ( 25)	4 ( 80)
**CCI^b ^Category **(n = 286)					
Low	240 (84)	142 ( 88)	86 (84)	12 ( 75)	0 ( 0)
Moderate to high	46 (16)	20 ( 12)	17 (16)	4 ( 25)	5 (100)
	**Mean (Sd)**	**Mean (Sd)**	**Mean (Sd)**	**Mean (Sd)**	**Mean (Sd)**
CCI^b ^score	0.7 (1.0)	0.6 (0.8)	0.8 (1.1)	0.8 (1.5)	2.4 (0.9)
Number of Co-morbidities Listed	2.5 (1.8)	2.4 (1.7)	2.5 (1.9)	2.6 (2.2)	5.2 (1.9)

^a^Where missing responses, percentages were calculated out of available responses

^b^CCI = Charlson Co-morbidity Index

Fifty-five percent (n = 160) of respondents had contracted the virus through anti-D, 8% (n = 22) via blood products and 33% (n = 96) by blood transfusion. The remaining 4% (n = 12) contracted the virus through alternative means which included a combination of sources (e.g., anti-D and blood transfusion, blood products (i.e., no conclusive source), blood products in a transplanted liver, mother-to-child transmission (n = 2, 0.6%)).

### Co-morbid Disease and Clinical Symptoms

Approximately 89% (n = 255) of the cohort reported at least one concurrent medical condition. Table 2 provides details of the most frequently specified disorders. Co-morbid illness was reclassified using the CCI to facilitate further analysis of the impact of co-morbid disease. The mean score for the total group was 0.7 ± 1.0 with a range of 0-6. The results of the CCI were then classified as pertaining to low (scores 0-1) or moderate to high (scores 2-6) co-morbidity levels. Eighty-four percent (n = 240) had low levels of co-morbidity while the remaining 16% (n = 46) were classified in the moderate to high category. Using a simple count method of co-morbidity measurement, the median listed number of co-morbidities per person was 2 and ranged from 0-9 (mean = 2.5 ± 1.8).

Self-report of symptoms highlighted important features of the condition. Table 2 illustrates the most frequent symptoms cited. These symptoms were categorised according to body structure involvement as hepatic and extra-hepatic symptoms, but were not mutually exclusive (62% (n = 169) and 99% (n = 275) respectively). Cacoub et al. [19] classified any symptoms involving joint, muscles and skin as extra-hepatic clinical manifestations while Poynard et al. [16] included fatigue among this group. The current study further distinguished symptoms of more advanced disease such as disturbed sleep [50] or abnormal renal function [51,52] as extra-hepatic. Analysis of these symptoms (Additional file 1) revealed that a greater proportion of females had hepatic symptoms (p = 0.007) and extra-hepatic symptoms (p = 0.004) than males. When the relationship with age was explored no significant association was noted. Virus positivity also failed to demonstrate any significant associations with either symptom. Moderate to high co-morbidity as measured by the CCI was associated with a higher prevalence of hepatic symptoms (p = 0.024). The presence of a mood disorder was significantly associated with greater hepatic symptoms only (p ≤ 0.001) while haemophilia was not significantly related to the presence of either symptom cluster. Analysis of the effects of the duration of infection on symptom prevalence revealed a significant association with longer durations of infection associated with the presence of extra-hepatic symptoms (p = 0.005).

### Pain and Fatigue

Pain and fatigue were examined further given that these symptoms are frequently cited in the literature as being the most common extra-hepatic symptoms of hepatitis C. Both fatigue and pain were present in 88% (n = 241) and 96% (n = 243) of the sample. Further exploration of pain and more specifically musculoskeletal pain (71%, n = 173) revealed multiple and varying sites of body pain. Breakdown of the location of this symptom found the most common sites were lower limb (73%), upper limb (67%), back (62%) and neck (28%). Fifty-seven per cent reported taking medication for this pain.

No significant relationship was found between presence of pain and age-group. Gender analysis on the other hand, showed that more females reported pain than males (p < 0.01). With regard to fatigue, there was a significant difference between age groups in prevalence rates for fatigue (p < 0.01) and females were more likely than males to experience fatigue (p = 0.011) (Additional file 1). Virus status was not significantly related to the presence of these symptoms, while the presence of a mood disorder was linked with symptoms of fatigue (p = 0.004). An association was noted between the presence of pain and fatigue though it did not reach statistical significance (χ^2 ^= 5.2; df = 1; p = 0.056, 95% CI = 0.7-26.3%).

### Factors Predicting Symptoms

Standard enter logistic regression analysis was performed for hepatic, extra-hepatic, pain and fatigue symptom categories to determine which factors were of clinical relevance. All variables presented for univariate analysis were entered into the multivariate analysis, Additional file 1 details each of the models. For hepatic symptoms; female gender (p = 0.039, OR 3.7), mood disorder (p ≤ 0.001, OR 7.5) and moderate to high co-morbidity (p = 0.022, OR 3.7) emerged significant predictors of having hepatic symptoms. When the duration of infection was removed from the model age under 50 years was also associated with the presence of hepatic symptoms (p = 0.024, OR 3.4). When the regression model was constructed for extra-hepatic symptoms no factor emerged as a significant independent predictor. Female gender and age group were both significant for fatigue. The under 50 years group had a higher odds (OR 19.1, p = 0.013) of reporting symptoms of fatigue than the other 2 groups. For pain, female gender was the sole significant predictor of this symptom in this cohort.

## Discussion

This study examined the main socio-demographic and clinical features of an iatrogenically acquired Irish hepatitis C cohort. A response rate of 28% while low, given the methodological limitations required by the patient advocacy groups for this postal survey to ensure anonymity, was considered a representative sample with nationwide response. Lack of direct access to patient databases affected the researchers control over recruitment of subjects and the ability to follow-up non-responders. The study cohort was subject to a gender and age bias given the population under review, but was reflective of the recently published report on the Irish iatrogenic HCV cohort from the National Hepatitis C Database[7]. The low response rate and the obvious gender imbalance however should still be considered when interpreting the results of this survey, in particular the regression analysis in view of the predictive effects of female gender in symptom experience.

Restricted access to the patient cohort necessitated use of self-report data, derived from both an open-ended and checklist-type question, for describing morbidity and clinical manifestations of disease. Medical chart or physician evaluated morbidity is often seen as "gold standard" however there is a growing body of research for the utility of self-reported data for predicting disability and survival [53-56]. Given the nature of the data retrieved respondent and recall bias were other factors to consider when interpreting the results presented. The absence of an age-matched control group, to compare symptoms and co-morbid disease prevalence, in a case-control design, limited the interpretation of results with regard to cause and effect. However the selection of a single sample cross-sectional method was justified in the planning stages of the current study given that prior published research on this area in general used this methodology. Only two studies were identified by the researcher that compared symptoms experienced by controls with the HCV group. Seeff et al. [57] showed significant greater prevalence of tiredness (37% v 26%, p = 0.01) and anorexia (17% v 5%, p = 0.0003) in HCV. Meanwhile, Hoofnagle [15] in their cohort (n = 108) when compared to healthy blood donors demonstrated a striking similarity in prevalence rates for fatigue (62% v 70%), however abdominal pain (31% v 17%), itching (32% v 21%), nausea (19% v 16%) and dark urine (23% v 5%) were more common in HCV patients.

As expected, the demographic profile reported shows ageing of the iatrogenic HCV cohort since previous studies in the late 1990s and given the well publicised mechanism of infection via anti-D, was dominated by middle-aged females in keeping with the National Hepatitis C Database findings [7]. These results are in stark contrast with other international studies with mixed transmission routes whose samples were younger and male dominated [20,21,58]. Previous studies completed on the Irish cohort of transfusion-associated hepatitis C [8,12,38,59] correspond well in terms of age profile when the time elapsed is taken into consideration. The unemployment rate (60%, n = 57) was high in those who were eligible to participate in the labour force by comparison to the general Irish population average which was 4.2% [60]at the time of the survey. Analysis of the reasons given for unemployment status revealed that 91% (n = 52) (55% of eligible active labour force participants), were unable to work due to permanent sickness or disability. This is particularly high given that the general Irish population statistics show that of the active labour force population with any disability or with disability due to chronic illness only 15.3% and 11% were unemployed respectively [61]. Based on these figures unemployment for those citing permanent sickness or disability was considerably higher among the iatrogenic HCV cohort than the general Irish population (55% v 15.3% and 11%).

The uniqueness of this study cohort, made comparison with results from other studies difficult due to mixed clinical, social, geographic and ethnic variables of international cohorts. Forty-three percent (n = 125) of the study sample were virus positive, a lower proportion than reported in previous studies of this Irish group, where medical chart review was performed [8,12] although this correlated well with the more recent data presented in the baseline report of the HPSC [7]. However, 34 respondents (11.7%) reported that they were now virus negative following treatment, bringing the historic virus positive profile of the group into concurrence with original studies where 55% of patients were virus positive [8]. The high proportion of the study cohort infected through a single source influenced the genotype profile reported (71% genotype 1) however this was consistent with the National Hepatitis C Database. Discrepancies noted between the current survey respondents and previous Irish iatrogenic cohorts [12,59] can be rationalised by virtue of the inclusion of both genders and all modes of infection. At the time of this study a mere 1% reported undergoing current treatment for HCV. The limited numbers of respondents treated (12% of the study cohort) and undergoing treatment may be indicative of limited treatment efficacy especially given the high proportion of the cohort with genotype 1 (most resistant to treatment) [62] but also the mildness of the disease progression in this population.

Approximately 99% (n = 275) of respondents had symptoms perceived as relating to their hepatitis C. This figure was notably higher than that reported in Kenny-Walsh's Irish study in 1999 [8] where 81% had symptoms. Cacoub and colleagues [19] reported the presence of at least one extra-hepatic manifestation in 74% of their study participants. Extra-hepatic symptoms were more prevalent (99%) in the current study than hepatic symptoms, which were reported in 62%. Differences in the levels of symptoms recorded in the current study were also noted when compared to the National Hepatitis C Database. This may be related to their non-specific nature and the different methods used to collate data. The National Hepatitis C Database [7] examined extra-hepatic manifestations in terms of biologically and clinically diagnosed phenomena (10.2%). Meanwhile the current study findings were based on self-reported symptomatology and not definitive diagnoses. The most prevalent symptoms here were fatigue and pain with 88% and 96% complaining of these symptoms respectively whereas in the Kenny-Walsh study of the anti-D cohort, 66% reported fatigue and 38% arthralgia. Similarly lower rates of fatigue (33-39%) and pain (23-25%) were reported in earlier studies in the Irish iatrogenic HCV population by Barrett et al. [11] and Coughlan et al. [12]. These high symptom prevalence rates (extra-hepatic 99%; pain 96%; fatigue 88%) are important to acknowledge as being open to possible respondent bias. However, advancing age and longer duration of infection at the time of the current study may also explain this increase. The use of a medical chart review in the National Hepatitis C Database [7] limited comparisons with this report, although the recorded levels of fatigue (30%) and arthralgia (24%) were substantially lower than the data presented here.

Cacoub et al. [19] found that the most frequent risk factors for the presence of clinical and biologic extra-hepatic manifestations were age, female gender and extensive liver fibrosis. Further analysis on the effects of age, gender, virus status and duration of infection on the presence of symptoms in the current cohort found that female gender was a significant predictor. Surprisingly no factor emerged as independently predictive of the combined category of extra-hepatic symptoms in this study. Closer examination of the two most common extra-hepatic complaints, fatigue and pain, revealed that female gender was a significant factor in both of these symptoms while age was an important predictor of fatigue only. Complaints of fatigue were less likely in older individuals in contrast to the findings of Cacoub et al. [19]. Virus status (chronic or self-limited infection) or duration of infection were not associated with the presence of symptoms though lack of knowledge of disease severity in this group limited further comparison with Cacoub's study. The lack of association of these clinical factors with pain and fatigue concurred with the findings of Barkhuizen et al. [45], who found that these symptoms while frequently present in patients with chronic hepatitis C were unrelated to the severity of the liver disease. The presence of symptoms in the absence of organic disease for the virus negative group is an interesting finding and concurred with earlier research in a similar cohort [11]. Symptoms in those with self-limited or resolved HCV infection may be related to physical or psychosocial influences associated with past infection with HCV. This requires further investigation. However, it should be noted that a high proportion (36%, n = 104) in the present study cohort failed to report their current viral status, therefore the relationship between symptoms and virology must be interpreted with caution here. The high levels of missing data may reflect a lack of knowledge in the cohort or may be due to omission or respondent selection. Meanwhile, similar to fatigue, associations with younger age were shown for the hepatic cluster of symptoms when duration of infection was removed from the regression model. No research has been conducted to date on factors predicting hepatic symptoms thus limiting comparisons.

In the current study 89% of the sample had at least one diagnosed medical condition. Recoding of the CCI illustrated that in the main the iatrogenic HCV cohort experienced low levels of co-morbidity (84%). The most frequent conditions reported were cardiovascular (50%), mood-related (48%) and musculoskeletal (31%), in keeping with other international studies [21,22,63]. The limited study in this area in the Irish cohort revealed some interesting figures for comparison with previously published data. When examining specific conditions, Kenny-Walsh [8] found the level of cardiovascular disorders was 8% and psychological 7%. The presence of co-morbidity has thus expanded vastly in this cohort. The presence of co-morbid disease is important to the overall well-being of this group as has been proven in previous studies, the amelioration of which may lead to improved HRQoL [20-22]. Reference figures for the National Hepatitis C Database [7] showed an underreporting of some co-morbid diseases e.g., depression (27%) by comparison with self-reported data in the current study (43%). Missing data and a lack of standardised medical record keeping across the different hepatology units was cited as a possible source of error in the National Hepatitis C Database hence the findings of this study may present a more representative picture of co-morbidity in this HCV population. Psychiatric illness as well as adversely effecting HRQoL, also plays a role in treatment success, given the importance of adherence to treatment which can be complicated by conditions such as depression. Mood disturbance and moderate to high co-morbidity was associated with hepatic symptoms thus supporting the premise that co-morbid disease is an important factor in health status for this group. Depression has been associated with stigma, well-being and adverse expectations of illness [24,40]. Dwight et al. [23] examined the relationship of depressive symptoms to fatigue and functional disability. They found that the severity of depressive symptoms highly correlated with fatigue severity, functional disability and somatisation. Proactive management of depression has been advocated to improve HRQoL and the efficacy of antiviral therapy for hepatitis C [58]. Co-morbid mood disturbance did show a significant association with extra-hepatic symptoms, fatigue in particular in this group though it was not an independent predictor of these symptoms. Further investigation and management of this symptom and mood disorders are warranted in this cohort given its' unclear patho-physiology and that McDonald et al. [64] and Dwight et al. [23] reported that fatigue was more closely related to psychopathology than hepatic disease.

The unlikely association of younger age and symptoms warranted further exploration of study findings. The haemophilia group although younger was predominantly male and had an equivalent duration of infection to the other patient groups. The combination of male gender and mean disease duration of 26 years could potentially be associated with faster disease progression [16,17,35,62,65,66] and hence higher symptom prevalence. Signs of decompensated liver disease include fatigue [67] and thus more information on liver disease severity would facilitate better interpretation of these results. No significant difference was found however between the groups in terms of symptom prevalence or mood disturbance (unpublished findings) which is important given that somatisation has been linked with mood disturbance in other studies [23,24]. Mood disorders were not associated with age (unpublished findings). The generally older age profile of the cohort meant that a significant proportion carried an additional disease burden (advanced age was associated with co-morbid disease p < 0.05). Those with renal disease and haemophilia had a higher percentage of the moderate to high category of co-morbid disease and this may have influenced study findings. Disease labelling and awareness of diagnosis has been associated with deficits in well-being [31]. The longer duration of awareness of diagnosis in the haemophilia group may have affected the patients' perception of symptoms. Indeed the complaints of fatigue and other symptoms usually associated with an older population may have been perceived more in the younger cohort where these symptoms were seen as abnormal and this may have affected the results [68].

Given the proven influence of viral and host factors in disease progression, the emerging role of co-morbidity in fibrogenesis, as is true for diabetes [17,66,69,70], and the need for these factors to be taken into consideration when deciding on suitability for antiviral treatment [67,71] further emphasises the need for implementing appropriate management strategies. The median time to cirrhosis development has been reported as 30 years [17], a time-frame within which the majority of this group were infected. With the development of cirrhosis, disease severity is progressing hence the need for effective interventions to influence the modifiable factors for disease progression such as weight, diet, alcohol consumption by means of a health promotion and activity programme. The high incidence of musculoskeletal and cardiovascular disorders highlights a potential rehabilitation and health promotion need in this cohort. A previous review into the proposed benefits of exercise in chronic liver disease found some support in the literature. It was suggested that a structured exercise programme could be used to complement the medical management strategies for hepatitis C, through the reduction of diabetes and cardiovascular risk, prevention of obesity and management of fatigue as well as musculoskeletal and neuropathic symptoms [72].

## Conclusion

Socio-demographic and clinical data characteristics presented here provide additional data to that recorded in the National Hepatitis C Database report, on an ageing iatrogenically infected HCV cohort in Ireland, which has relevance for future planning of hepatology services. The growing disease burden of the Irish iatrogenic HCV cohort represented here by comparison to previous research conducted in this population emphasises the importance of prospective follow-up to document the emerging symptoms and co-morbid disease and indeed the suitability of this unique group for follow-up. Much has been written in the literature about the effects of ageing on disease progression. International research has highlighted an expected "age wave of disease burden" with chronic hepatitis C [67]. The advancing age and associated shift in co-morbid disease prevalence identified here has important implications for cirrhosis development and advancement for this cohort. In addition to influencing liver disease progression, these co-morbid diseases may also independently influence healthcare utilisation and QoL. The emergence of high levels of disease-related symptoms is another important factor to consider when planning future health services for this cohort. Higher disease burdens were identified in those under 50 years of age, females and those with co-morbid physical and psychological conditions and thus strategies need to be developed to target these vulnerable sub-groups. Review of existing and the development of new appropriate health policies and management strategies for HCV are essential to address the long-term care of this cohort and will inform the health provision for HCV patients of all infection routes.

This study showed a growing healthcare burden reported by HCV sufferers, emphasising the myriad of effects of the HCV in terms of diffuse symptoms and associated co-morbidity. The high prevalence and nature of the co-morbidities reported combined with the frequent complaints of pain and fatigue, indicate the need for strategies which focus more on physical health and health promotion. Health service planners and practitioners need to target and direct resources to the management of disease-related symptoms and prevention of co-morbidity specifically, thus addressing the additional healthcare needs more effectively given the current limited antiviral treatment success.

While the response rate was low, the study cohort was nonetheless representative of the Irish iatrogenic HCV population. The results presented provide data regarding the evolving health and social circumstances of patients with iatrogenically transmitted hepatitis C infection in Ireland, adding to existing knowledge regarding the clinical course and consequences of this source of HCV infection over time.

## Competing interests

The authors declare that they have no competing interests.

## Authors' contributions

OMcK and CB both contributed equally in the design and implementation of the study. OMcK collected the data, performed the statistical analysis and prepared the manuscript. CB gave statistical expertise and revised the manuscript for intellectual content. CC assisted in interpretation of data analysis and drafting of the manuscript. All authors read and approved the final manuscript.

## Pre-publication history

The pre-publication history for this paper can be accessed here:



## Supplementary Material

Additional file 1 **Iatrogenic Hepatitis C - Factors Associated with Symptoms ** Click here for file
