# Supplementary material for: Socio-demographic and clinical features of Irish iatrogenic hepatitis C patients: a cross-sectional survey
Source: BMC Public Health. 2009 Sep 7;9:323. doi: 10.1186/1471-2458-9-323 (PMC2761402; doi:10.1186/1471-2458-9-323)
Supplement: Additional file 1 — Iatrogenic Hepatitis C - Factors Associated with Symptoms [file 1471-2458-9-323-S1.doc]

| **Table 3 Iatrogenic Hepatitis C - Factors Associated with Symptoms** | | | |  |  |  |  |
| --- | --- | --- | --- | --- | --- | --- | --- |
|  | **Age Group^c^ (n=281)**  <50 (n=69) 51-60(n=132) 61-80 (n=80) | **Gender (n=281)** Male (n=44) Female (n=237) | **Virus Status (n=186)** Positive (n=125) Negative (n=61) | **Mood Disorder (n=286)** Present (n=136) Absent (n=150) | **Haemophilia (n=286)** Present (n=19) Absent (n=267) | **Categorised CCI ^b^Score (n=286)** Low (n=240) Moderate to high (n=46) | **Duration of Infection (Years)** |
|  |  |  |  |  |  |
|  | **n (%)^a^** | **n (%)^a^** | **n (%)^a^** | **n (%)^a^** | **n (%)^a^** | **n (%)^a^** | **Mean (Sd)** |
| **Hepatic (n=169)** | 41 (63) | 19 (44) | 79 (63) | 102 (79) | 9 (47) | 135 (59) | 26.1 (5.6) |
|  | 82 (65) | 150 (66) | 41 (68) | 67 (47) | 159 (63) | 34 (77) |  |
|  | 46 (58) |  |  |  |  |  |  |
| **Univariate Analysis**  χ2 (df)/ t (df) 95% CI | n/s* | **p=0.007*** 7.2(1) 5.6-36.5 | n/s* | **p≤0.001*** 29.9 (1) 20.9-42.3 | n/s* | **p=0.024*** 5.1 (1) 2.5-30.0 | n/s*** |
| **Multivariate Analysis Odds Ratio** | n/s | **p=0.039 3.7** | n/s | **p≤0.001 7.5** | n/s | **p=0.022 3.7** | n/s |
| **Extra-hepatic (n=275)** | 65 ( 99) | 40 (93) | 123 (98) | 132 (100) | 19 (100) | 228 ( 99) | 26.5 (5.3) |
|  | 130 (100) | 232 (100) | 60 (98) | 141 ( 98) | 253 ( 99) | 45 (100) |  |
|  | 77 ( 98) |  |  |  |  |  |  |
| **Univariate Analysis**  χ2 (df)/ t (df) 95% CI | n/s* | **p=0.004**** 16.4(1) 2.1-18.6 | n/s** | n/s* | n/s** | n/s** | **p=0.005*****  2.9 (251) 2.7-14.9 |
| **Multivariate Analysis Odds Ratio** | n/s | n/s | n/s | n/s | n/s | n/s | n/s |
| **Pain (n=243)** | 56 (95) | 29 (85) | 109 (97) | 120 (97) | 15 (100) | 201 (96) | 26.3 (5.1) |
|  | 120 (97) | 211 (98) | 53 (98) | 121 (96) | 225 ( 96) | 40 (98) |  |
|  | 64 (97) |  |  |  |  |  |  |
| **Univariate Analysis**  χ2 (df)/ t (df) 95% CI | n/s* | **p=0.003**** 13.9(1) 4.1-28.3 | n/s** | n/s** | n/s** | n/s** | n/s*** |
| **Multivariate Analysis Odds Ratio** | n/s | **p=0.003**  **31.6** | n/s | n/s | n/s | n/s | n/s |
| **Fatigue (n=241)** | 61 (94) | 33 (77) | 112 (90) | 121 (94) | 15 (79) | 198 (87) | 26.4 (5.4) |
|  | 116 (91) | 206 (90) | 53 (88) | 118 (83) | 223 (89) | 41 (93) |  |
|  | 62 (79) |  |  |  |  |  |  |
| **Univariate Analysis**  χ2 (df)/ t (df) 95% CI | **p=0.006*** 10.4 (2) | **p=0.011*** 6.4(1) 2.5-28.4 | n/s* | **p=0.004***  8.1 (1) 3.6-19.0 | n/s** | n/s* | n/s*** |
| **Multivariate Analysis Odds Ratio** | **p=0.013 19.1** | **p=0.009 8.5** | n/s | n/s | n/s | n/s | n/s |
| * Chi Square ** Fishers Exact, *** Independent t test. ^a^ Where missing responses, percentages were calculated out of available responses, ^b^ CCI - Charlson Co-morbidity Index, ^c^ No 95% Confidence Interval (CI) calculated due to the presence of 3 variables. | | | | | | | |
